# Contribution of Insoluble Bound Antioxidants from Germinated Seeds of Wheat and Spelt to the Nutritional Value of White Bread

**DOI:** 10.3390/molecules28176311

**Published:** 2023-08-29

**Authors:** Marjeta Mencin, Katja Golob, Maja Krek, Tomaž Polak, Tomaž Požrl, Petra Terpinc

**Affiliations:** Department of Food Science and Technology, Biotechnical Faculty, University of Ljubljana, Jamnikarjeva 101, SI-1111 Ljubljana, Slovenia; marjeta.mencin@bf.uni-lj.si (M.M.); tomaz.polak@bf.uni-lj.si (T.P.); tomaz.pozrl@bf.uni-lj.si (T.P.)

**Keywords:** germination, spelt, wheat, bread, functional food, phenolic antioxidants, LC-MS, ferulic acid, *p*-coumaric acid

## Abstract

This research aimed to study the dynamic changes in phenolic antioxidants between the germ and the rest of the germinated seed of wheat and spelt and to evaluate the whole grain flour from germinated seeds as a functional supplement. Longer germination resulted in higher TPC, DPPH, and ABTS values when considering the entire germinated seed, while the optimal germination time was not consistent when considering the germ and the remaining germinated seed separately. While in the germinated seed (without germ) the majority of TPC was determined to be bound phenolics (up to 92%), the extractable form dominated in the germ (up to 69%). The most abundant phenolic antioxidants in germinated wheat and spelt seeds, *trans*-ferulic acid, *cis*-ferulic acid, and *p*-coumaric acid, increased significantly with germination. Only breads with 5% germinated spelt or wheat flour were suitable for the production of a food product, showing higher extractable TPC, antioxidant activity, individual phenolic acids, and improved specific volume, and were preferred because of their appearance, aroma, and color. The PCA biplot showed that the addition of 15% and 30% germinated flours had the greatest positive impact on phenolic properties, while breads with the addition of 5% germinated flour had the greatest positive impact on specific volume and color.

## 1. Introduction

Several epidemiological studies have established a link between the consumption of dietary polyphenols and a reduction in the risk of developing some chronic diseases, which has led consumers to search for healthier, natural, polyphenolic/antioxidant-enriched foodstuffs [1]. Research indicates that cereal grains contain special phenolic compounds, such as ferulic acid and diferulates, which are not present in significant quantities in fruit or vegetables. Wheat is the main source of cereal products, but they are mostly produced from refined white flour from which peripheral tissues have been removed [2]. Clinical studies on obesity comparing the effect of whole grain flour and white flour have shown that the preference of the former might be attributed to the ability of dietary fiber to reduce the digestibility of high-caloric molecules (fat and sugars) in the small intestine and to induce faster satiety [3]. Spelt is a hexaploid series of the *Triticum* genome constitution, which is characterized by great adaptation to a wide range of environments. Although many studies support the assumption of a healthier nutritional and functional potential of ancient wheat, other in vitro studies claim that it is not possible to definitively conclude that ancient wheat varieties are superior to all modern varieties in reducing the risk of chronic diseases [4]. Bread is the most important baked wheat product and a staple food worldwide [3], and therefore can be considered to be an optimal medium for functional supplements. These functional components can originate from germinated cereals since nutritional changes during seed germination occur [5]. Moreover, our recent studies indicated that germination of spelt seeds affects the quantity, form, and accessibility of phenolics present [6,7,8,9,10].

Non-extractable phenolics bound to dietary fiber are an interesting source for the food industry, since dietary fiber and antioxidants are recognized as health-promoting ingredients with nutritional claims approved by the European Food Safety Authority (EFSA), such as “high fiber”, as well as having technological advantages (e.g., increased storage stability, improved water- and fat-holding capabilities, or functioning as a non-caloric bulking agent), leading to new products with added value [1]. Although phenolics are not required for vital body functions in humans, they have become of great interest due to their anti-allergenic, anti-artherogenic, anti-inflammatory, antimicrobial, antioxidant, antithrombotic, cardioprotective, and vasodilatory functions. There is a great deal of evidence for their beneficial physiological effects, such as the inhibition of cell cancer proliferation, protection to neurons, improvement in insulin secretion, reduction in vascularization, and stimulation of vasodilatation [11]. 

The objectives of the present study were (1) to investigate dynamic changes in the extractable and bound phenolics contents and their antioxidant activities separately in germs and the corresponding remaining wheat and spelt seeds; (2) to monitor the content of ferulic and *p*-coumaric acids in extractable and bound forms at different germination stages; and (3) to evaluate the effect of supplementation of white flour with whole grain flour from germinated wheat or spelt seeds on sensory and nutritional characteristics of experimental functional bread.

## 2. Results

### 2.1. Non-Germinated Seeds vs. Germinated Seeds

The TPC, DPPH, and ABTS results of purified extracts of non-germinated and germinated seeds are presented in Table 1. After 48 h, germ on average represented 3.3 and 4.1% of total seed dry weight (DW), for wheat and spelt, respectively; after 96 h of germination, the proportion increased to 11.3 and 14.7%. Minor differences were observed between the reactivity of spelt and wheat extracts within the individual assays for non-germinated seeds (Table 1). In order to compare non-germinated seeds with germinated seeds, the results for germinated samples were expressed as the sum of the contents determined for the germ and the rest of the seed, taking into account their DW contribution to the whole part. After 48 h, extractable TPCs increased 5.7-fold and 4.4-fold for wheat and spelt, respectively, while after 96 h, the increases were 14.1-fold and 12.4-fold, when compared to the non-germinated seeds. On the other hand, a slow increase (approx. 13.5%) in the content of the bound fraction after the first 48 h of germination was observed for both species, while after the next 48 h (96 h germination), it increased 1.3-fold for wheat and 1.6-fold for spelt, when compared to non-germinated seed. With regard to the DPPH assay, the reactivity of extractable fraction after 48 h was increased 4.7-fold and 5.1-fold for wheat and spelt, respectively, while even better results were obtained after 96 h (9.7-fold and 14.3-fold, respectively). In the case of bound antioxidants, a maximum 1.9-fold increase was observed, namely for spelt after 96 h. Extractable ABTS^•+^ scavenging activity of wheat and spelt after 48 h increased by 5.6-fold and 3.2-fold, respectively, and 12.2 and 7.9-fold, respectively, after 96 h of germination. For bound antioxidants, a max 2.0-fold increase in their reactivity toward ABTS^•+^ radicals was observed for spelt after 96 h. Despite the relatively low increase in bound phenolics/antioxidants in comparison to extractable ones during germination, it should be stressed that they still represented the majority of total phenolics.

#### 2.1.1. TPC

**Figure 1:**
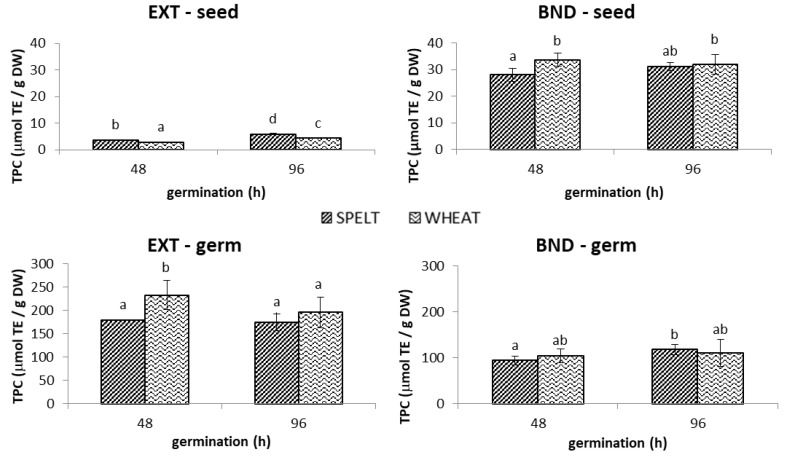
The content of extractable (EXT) and bound (BND) phenolics in the spelt and wheat seed and germ germinated 48 h and 96 h was determined by the Folin–Ciocalteu (TPC) assay. Data are means ± standard deviation, and different letters indicate significant differences (*p* < 0.05; LSD post hoc test).

Germinated spelt seed (without germ) contained higher extractable phenolics after 48 h (by 26%) and 96 h (by 35%) of germination than wheat seed (Figure 1) but regardless of the *Triticum* species, a longer germination time resulted in higher TPC. Different results were obtained for the bound fraction, whereby the highest TPC content was determined for wheat after the first 48 h (33.7 µmol/g DW), and the value did not differ statistically from spelt germinated for 96 h. While the majority of TPC in the seed was determined as bound phenolics (up to 92%), the extractable form dominated in the germ (up to 69%). In relation to the germ, the highest extractable TPC was determined in wheat after 48 h (233.2 µmol/g DW), which was one-third more than that in spelt. In terms of bound TPC, there was no significant difference between the germs of the two species, but it should be stressed that the levels were significantly lower than their corresponding extractable fractions (Figure 1).

**Figure 2:**
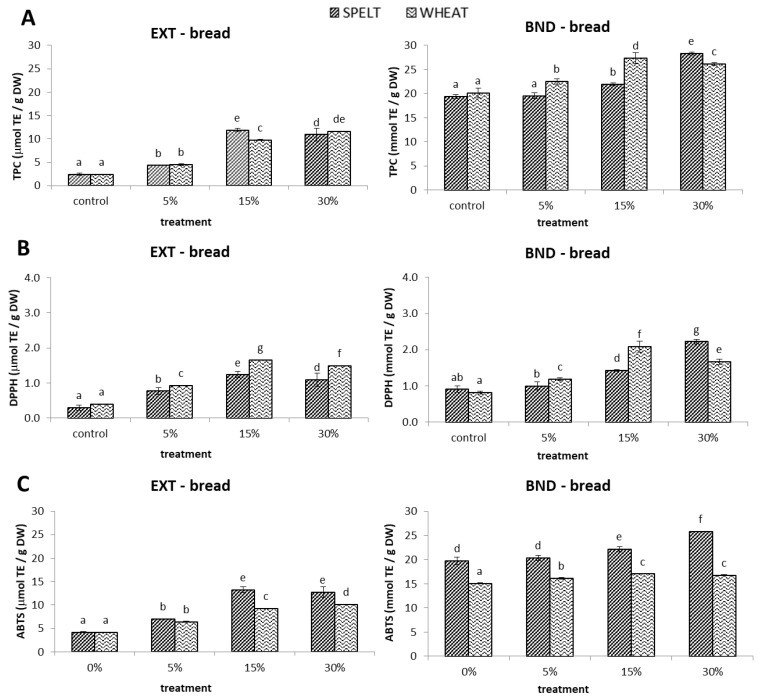
Total phenolic content (TPC) (**A**) and in vitro antioxidant activities (DPPH^•^ (DPPH) (**B**) and ABTS^•+^ (ABTS) (**C**) antioxidant capacity) for the extractable (EXT) and bound (BND) phenolics in the breads with 0%, 5%, 15%, and 30% added spelt or wheat germinated flour. Data are means ± standard deviation, and different letters indicate significant differences (*p* < 0.05; Duncan’s Multiple Range Test).

In the bread test, we found that the extractable TPCs of control wheat and spelt breads were 2.4 µmol/g DW, while bound TPCs were about 20 µmol/g DW, but values did not differ statistically between control breads (Figure 2A). Even the slightest addition of germinated spelt or wheat flour to bread contributed to an increase in extractable TPCs of 79% and 87% compared to the control, respectively. The highest significant increase in extractable TPC was observed in “bread S15” (389%) and “bread W30” (379%) compared to the control breads. The addition of 5% and 15% germinated flours doubled the extractable TPC in breads. Despite the doubled allowance of germinated spelt flour (from 15 to 30%), no increase in extractable TPC was achieved. Moreover, at a 30% addition of germinated spelt flour, the extractable TPC was significantly reduced compared to “bread S15”. The addition of germinated flours increased the relative proportion of extractable TPC over total phenolics (extractable + bound) from 11% (control) to 35% (bread S15) (Figure 2). The highest increase in bound TPC was observed for “bread S30” (46%) and “bread W15” (36%). To summarize, the highest content of extractable TPC in bread was obtained by adding 15% flour from germinated spelt or 30% flour from germinated wheat, while it was the opposite for bound TPC.

#### 2.1.2. DPPH^•^ Antioxidant Capacity

**Figure 3:**
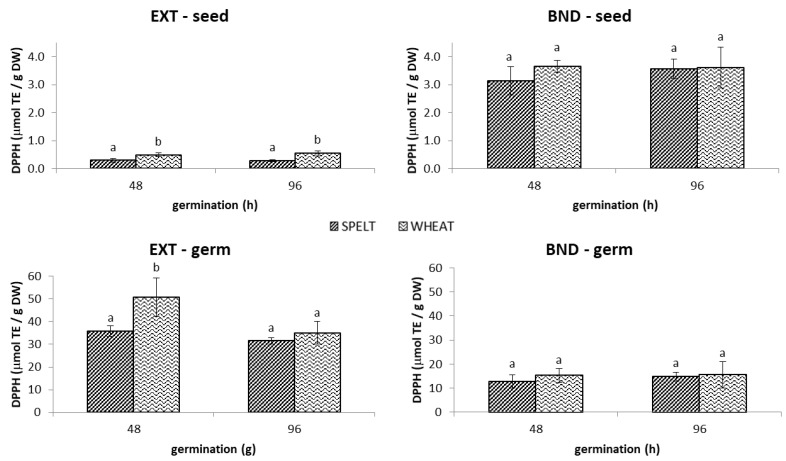
The DPPH^•^ antioxidant capacity of extractable (EXT) and bound (BND) phenolics in the spelt and wheat seed and germ germinated for 48 h and 96 h. Data are means ± standard deviation, and different letters indicate significant differences (*p* < 0.05; LSD post hoc test).

Extractable DPPH values remained constant after a 96 h germination period for spelt and wheat seed (without germ), whereby DPPH values of wheat were about 1.8-times higher than those of spelt and reached their maximum at 0.6 µmol/g DW (Figure 3). On the other hand, there was no statistical difference in bound DPPH values between two samplings (48 and 96 h) within the same *Triticum* variety, nor between the two varieties, with values reaching a maximum of 3.7 µmol/g DW. Similar to the case of TPC, the bound fraction represented up to 93% of all antioxidants in the remaining part of the seed after the germ was removed. It has to be stressed that considerably higher extractable DPPH values were obtained for germ, ranging from 64-fold (wheat 96 h) to 123-fold (spelt 48 h) higher values when compared to the remaining germinated seed. After 48 h, the bound antioxidants in germ represented only one quarter of all compounds capable of scavenging DPPH^•^ radicals; after 96 h of germination, their proportion increased to one-third. Similar to extractable antioxidants, bound phenolics of spelt and wheat also expressed comparable activity at both stages of germination.

As expected from the results obtained on the germinated samples, bread tests showed that wheat breads possessed higher extractable DPPH^•^ scavenging activities than spelt breads (Figure 2B). The highest increase in extractable DPPH values was obtained in “bread S15” (324%) and “bread W15” (313%) compared to the corresponding control breads. Interestingly, the addition of 30% of spelt and wheat germinated flours resulted in a decrease in extractable DPPH^•^ scavenging activities, by 11% and 10%, respectively, compared to the addition of 15% of germinated flours. The addition of 30% germinated spelt flour and 15% germinated wheat flour resulted in the highest increase in bound DPPH^•^ scavenging activity, of 147% and 154%, respectively, compared to the corresponding control breads. There were quite large differences in bound DPPH^•^ scavenging activity between spelt and wheat breads. The contribution of extractable DPPH antioxidants ranged from 33% in “bread S30” to 47% in “bread W30”, while in the control spelt and wheat breads, the extractable fractions accounted for 24% and 33% of all antioxidants. Both the variety and the percentage of germinated flour addition therefore had a significant impact on the obtained values.

#### 2.1.3. ABTS^•+^ Antioxidant Capacity

**Figure 4:**
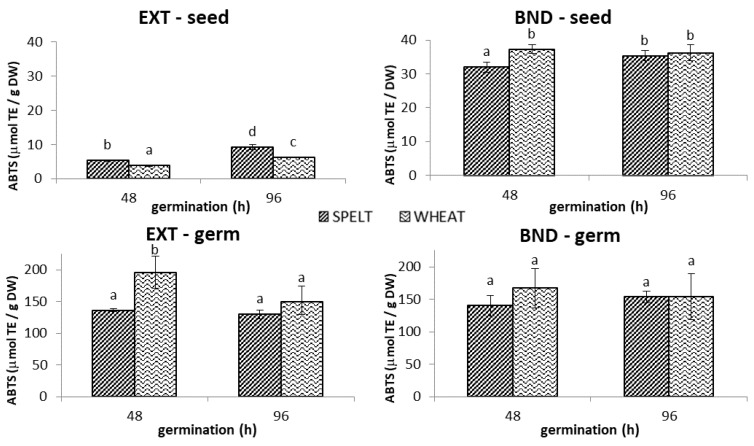
The ABTS^•+^ antioxidant capacity of extractable (EXT) and bound (BND) phenolics in the spelt and wheat seed and germ germinated for 48 h and 96 h. Data are means ± standard deviation, and different letters indicate significant differences (*p* < 0.05; LSD post hoc test).

As can be seen from Figure 4, prolonged germination led to a higher content of extractable antioxidants, whereby spelt seed expressed better activity (by 48%) after 96 h than did wheat seed. At the same time, at the end of the germination experiment, spelt and wheat seeds showed similar ABTS^•+^ scavenging efficiency of bound fractions, with values amounting to 79% in spelt and 85% in wheat, as that of total phenolics. In contrast, in the 96 h old germ, bound fractions were responsible for only half of the ABTS^•+^ scavenging activity (Figure 4). At early stages of germination, the extractable fraction from wheat germ expressed 44% better antioxidant activity than the corresponding sample from spelt, but its efficiency in the ABTS assay was comparable after 96 h. A considerably higher content of extractable antioxidants was observed in germ when compared to the remaining seed. The reactivity of bound antioxidants in the ABTS assay among analyzed *Triticum* germs followed the same trend as that in the DPPH assay, with neither the variety nor the germination time having a significant impact on the obtained values, which amounted on average to 154 µmol/g DW.

In the bread test, we observed higher absolute extractable and bound ABTS^•+^ scavenging activity in spelt breads than in wheat breads (Figure 2C). The highest increase in extractable ABTS^•+^ scavenging activity was obtained in “bread S15” (217%) and “bread W30” (147%) compared to the corresponding control breads. The contribution of extractable ABTS antioxidants to the total ranged from 26% in “bread S5” to 38% in “bread W30”, while in the control spelt and wheat breads, the extractable fractions accounted for 18% and 21% of all antioxidants, respectively. The highest increase in bound ABTS values was obtained in “bread S30” (30%) and “bread W15” (13%) compared to the control breads. The reactivity of bound antioxidants in the ABTS assay in spelt and wheat breads followed the same trend as in the DPPH assay (although the absolute concentrations were significantly higher). In particular, the variety had a significant impact on bound ABTS values, with spelt breads showing higher bound ABTS^•+^ scavenging activity than wheat breads.

### 2.2. Content of Ferulic and p-Coumaric Acids

#### 2.2.1. *p*-Coumaric Acid

Table 2 shows that germinated seed (after germ removal) contained around 2 µg/g DW of extractable *p*-coumaric acid, regardless of the germination time and *Triticum* variety analyzed. The germination period had a significant impact only on bound *p*-coumaric acid in spelt seed. The content of bound *p*-coumaric acid in germ increased by 175% in wheat and 105% in spelt in the last 48 h of germination. The bound form ranged from 16 µg/g in seed to 489 µg/g in germ (96 h).

The highest content of *p*-coumaric acid was found in “bread W15”, with 22 µg/g DW, which was 34% higher than in the control “bread W0” (Table 3). In general, the breads with germinated wheat flour had a higher extractable *p*-coumaric acid content than breads with germinated spelt flour. No difference in extractable *p*-coumaric acid content was observed between the control “bread S0”, and breads with 15% and 30% added germinated spelt flour. The addition of 5% germinated spelt or wheat flour showed no significant difference in bound *p*-coumaric acid compared to the corresponding control breads, while the addition of 15% and 30% germinated flour significantly increased the bound *p*-coumaric acid content. Bread enriched with 30% spelt or wheat germinated flour showed the highest increase, by 411% and 341%, respectively, compared to the corresponding controls. No difference was found between the addition of germinated spelt or wheat flour.

#### 2.2.2. *trans*-Ferulic Acid

From Table 2 it can be seen that *trans*-ferulic acid is the predominant phenolic acid in wheat and spelt seed and germ. Among germinated samples, the lowest content of extractable *trans*-ferulic acid was found in germinated seed (around 16 µg/g), with similar values being determined for spelt and wheat, regardless of the germination time. The extractable fraction represented only 4% of total *trans*-ferulic acid in germinated seed, in contrast to germ, in which its proportion increased to 13%. Extractable *trans*-ferulic acid was the highest in 48 h old wheat germ, which was not statistically significantly different from the content in 96 h old germ of spelt. The bound *trans*-ferulic acid content in wheat and spelt germ was more than 5-times higher than in the corresponding germinated seeds. 

The highest content of extractable *trans*-ferulic acid was found in “bread S5”, the concentration being 7-fold higher than in the control “bread S0” (Table 3). Any kind of addition of germinated wheat flour showed statistically the same increase in the content of extractable *trans*-ferulic acid compared to the control bread. For bound *trans*-ferulic acid, the addition of 5% germinated spelt or wheat flour showed no difference compared to the control breads, while the addition of 15% or 30% germinated flours showed a significant increase in bound *trans*-ferulic acid content, especially “bread S30”, with a 7-fold increase compared to the control bread. The majority of ferulic acid in breads was in bound form. The addition of 5% germinated spelt or wheat flour resulted in a decrease in the ratio of bound *trans*-ferulic acid to the total (extractable + bound), from 99% (“bread S0”) to 95% (“bread S5”), and from 96% (“bread W0”) to 92% (“bread W5”).

#### 2.2.3. *cis*-Ferulic Acid

A longer germination period led to minor but significant increases in *cis*-ferulic acid in spelt and wheat seeds (around 16 µg/g) (Table 2), but it did not have an impact on its bound form, which still represented around 91% of the total. On the other hand, in germ, extractable *cis*-ferulic acid reached almost 20% of the totals, and differences between spelt and wheat were observed in samples germinated for longer periods. For bound *cis*-ferulic acid, the amounts in germ were about 10-fold higher than in the corresponding germinated seed (Table 2), with differences found among the *Triticum* cultivars studied.

Interestingly, 15% of added germinated wheat flour showed the highest increase (180%) in extractable *cis*-ferulic acid content compared to the control “bread W0” (Table 3), although there were no significant differences in extractable *cis*-ferulic acid between breads with different percentages of germinated spelt and wheat flours. Bound *cis*-ferulic acid represented more than 96% of the total, an exception being “bread S5”, in which the bound form represented 93% of the total. Interestingly, the addition of 5% germinated spelt flour created no significant differences in bound *cis*-ferulic acid compared to the control bread. A higher addition (15% and 30%) of germinated spelt or wheat flour significantly increased the bound *cis*-ferulic acid content. No difference in bound *cis*-ferulic acid was observed between the addition of 15% germinated wheat flour and 30% spelt or wheat flours.

### 2.3. Specific Volume 

Table 4 shows specific volumes (mL/g) of bread samples. It was observed that only the addition of 5% germinated spelt or wheat flour significantly increased the specific volume of bread compared to the control breads, by 11% and 14%, respectively. It is worth noting that the specific volume of “bread W5” was one-third higher than that of “bread S5”. When 30% germinated spelt or wheat flour was added, the specific volume of the breads significantly decreased, by 32% and 29%, respectively, compared to the control breads.

### 2.4. Color Measurement

**Figure 5:**
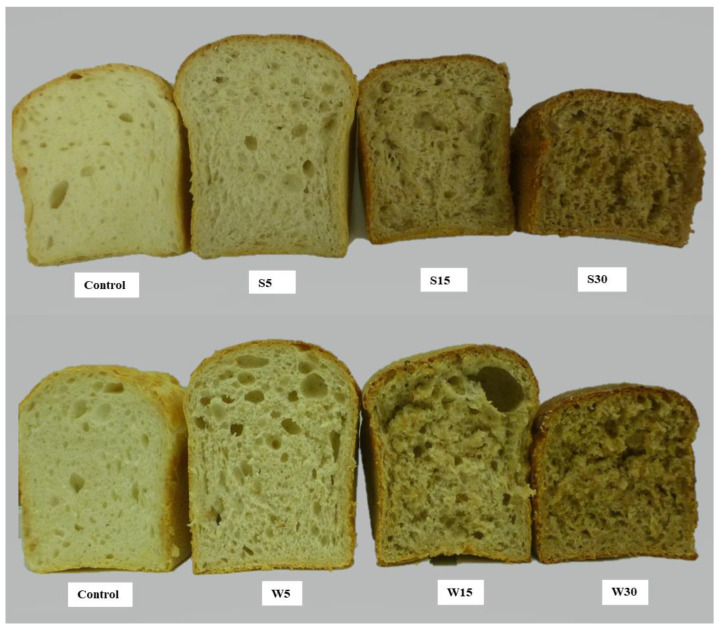
Cross-sections of baked breads formulated with germinated spelt (S) or wheat (W) flour. S0: control bread with no added germinated flour; S5: bread with added 5% germinated spelt flour; S15: bread with added 15% germinated spelt flour; S30: bread with added 30% germinated spelt flour; W0: control bread with no added germinated flour; W5: bread with added 5% germinated wheat flour; W15: bread with added 15% germinated wheat flour; W30: bread with added 30% germinated wheat flour.

The color parameters of the bread crust are listed in Table 4. Control “bread S0” and “bread W0” gave the highest luminosity (L*) values. The L* value of breads significantly decreased with the higher percentage of added germinated flour. The highest browning (100-L*) value was shown by breads with 30% added germinated spelt or wheat flour (Figure 5). The highest luminosity and lowest redness (a*) values of the bread color were obtained with control breads. Addition of germinated flour led to an increase in the a* and ΔE values of the breads. The ΔE values showed that the percentage of added germinated spelt or wheat flour had a significant impact on the total color change of breads. The addition of 30% germinated spelt or wheat flour decreased bread yellowness (b*) significantly, by 43% and 14%, respectively (Table 4). The percentage of added germinated flour significantly affected the b* value of breads and caused a huge decrease in the b* value, especially in the case of added germinated spelt flour.

### 2.5. Sensory Evaluation

Highly trained assessors with the necessary knowledge and experience in general descriptive analysis and with previous experience in evaluating bread participated in the assessment of the sensory attributes of the bread samples. They were asked to evaluate a slice of each bread for appearance, color, aroma, taste, texture and overall acceptability, using descriptive sensory analysis. Control “bread S0” and “bread W0” possessed a pleasant odor, taste, and aroma, good porosity, good shape, elastic-flexible crumb, a color typical of wheat bread, and a heterogeneous structure (no special features). “Bread S5” had a larger volume than the control bread, an uneven structure, more holes in the crumb, a good aroma, a slightly specific odor, an acceptable color, poor elasticity, and a slightly sticky crumb. “Bread W5” had also a larger volume than the control bread, a similar odor to “bread S5”, a mild specific odor, a little sticky crumb, poor flexibility, and unevenly distributed porosity. “Bread S15” and “bread W15” had a very sticky and wet crumb, and unacceptable quality, being sticky and lumpy in the mouth. The “bread W15” crumb was less bitter than “bread S15” and the crust had a bitter aftertaste. The aftertaste of roasted malt was too pronounced in “bread S15”. “Bread S30” and “bread W30” were unacceptable, having a very sticky and wet crumb, a caramel bitter taste, and a bitter taste even in the crumb, possibly due to strong proteolysis. These two breads were excluded from further sensory evaluation.

After one day, the sensory evaluation was repeated. The control breads did not show any special features. “Bread S5” was still a little sticky, which did not affect freshness since the stickiness sensation was predominant. On the other hand, “bread W5” had slight side odor, but it was pleasant in the mouth, not too sticky, and definitely better than the one-day-old “bread S5”. “Bread S15” was still very sticky and the addition of germinated spelt flour had no positive effect on prolonging freshness. “Bread W15” had uneven porosity, larger air holes in the crumb, and tunnels below the crust; the crumb was still very sticky but had no bitter aftertaste, while the crust had a slightly bitter aftertaste. According to sensory evaluation, it was found that, in terms of texture, flavor, taste, and overall acceptability, control breads with no added germinated flour were highly preferred, while in terms of appearance, aroma, and color, breads with a 5% addition of germinated flour were preferred. The addition of 15% and 30% of germinated spelt or wheat flour had a negative effect on the sensory properties of bread. The addition of germinated flour also had a significant influence on bread color acceptance according to the assessors (Figure 5). In general, the assessors preferred breads enriched with up to 5% germinated flour. 

### 2.6. Principal Component Analysis

**Figure 6:**
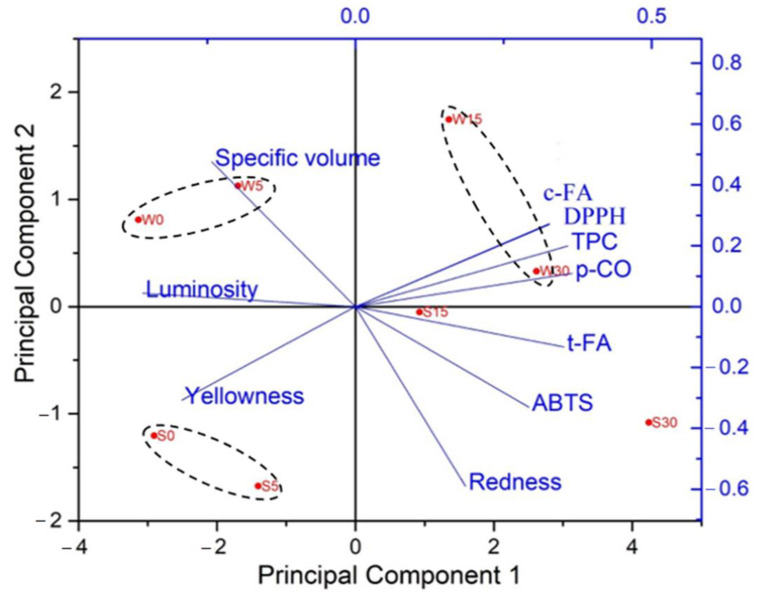
PCA biplot of the first two principal components from analysis of breads with added 5%, 15%, and 30% germinated spelt or wheat flour based on the contents of total *p*-coumaric acid (*p*-CO), *trans*-ferulic acid (t-FA), *cis*-ferulic acid (c-FA), total phenolic content (TPC), scavenging activities against DPPH^•^ (DPPH) and ABTS^•+^ (ABTS), specific volume, luminosity, yellowness, and redness. S0: control bread with no added germinated flour; S5: bread with added 5% germinated spelt flour; S15: bread with added 15% germinated spelt flour; S30: bread with added 30% germinated spelt flour; W0: control bread with no added germinated flour; W5: bread with added 5% germinated wheat flour; W15: bread with added 15% germinated wheat flour; W30: bread with added 30% germinated wheat flour.

Principal component analysis (PCA) is one of the most classical and widely used techniques for reducing dimensionality and increasing the interpretability of large datasets. Principal component 1 (PC1) explained up to 72.3% of total variance and PC2 explained 15.0%. The presented two-dimensional graph was thus able to explain 87.3% of the variability in the experimental data (Figure 6). Samples were separated along the first PC by differences observed in *p*-coumaric acid, *trans*-ferulic acid, *cis*-ferulic acid, TPC, DPPH, and luminosity. PC2 separated the samples based on specific volume, yellowness, ABTS, and redness. Figure 6 shows that the samples are divided into three groups according to the percentage and type of added germinated flour. It can be seen that the groups do not overlap, so a clear separation can be observed, especially between germinated spelt and wheat flours.

Breads with 15% and 30% added germinated spelt or wheat flour are positioned on the right side of the graph but, as can be seen from Figure 6, the separation of these breads is based on spelt or wheat flour and not on the percentage of added germinated flour. Breads with 30% added spelt flour have a significantly higher content of total *trans*-ferulic acid, better ABTS^•+^ scavenging activity, and a higher redness value. In contrast, breads with 15% and 30% added wheat flour have a significantly higher content of total *cis*-ferulic acid and TPC and have better DPPH^•^ scavenging activity.

It can also be seen that the control breads and bread with 5% added germinated spelt flour are positioned close to each other on the left side of the graph, suggesting some similarities in phenolic characteristics, color, and specific volume. They exhibited the highest luminosity and yellowness. The wheat control bread and bread with 5% germinated wheat flour are positioned in the upper-left quadrant, suggesting that they have the highest specific volume.

The PCA biplot shows that the addition of 15% and 30% germinated flours has the greatest positive impact on phenolic characteristics, while breads with 5% added germinated flour show the greatest positive impact on specific volume and color.

## 3. Discussion

### 3.1. Germinated Seeds and Germs

Whole grain cereals deserve more attention as a rich source of bioactive health-promoting components, which include several phenolic acids bound to the dietary fiber [12]. Bound phenolics have strong antioxidant activity and may modulate cellular oxidative status and prevent oxidation of DNA, proteins, and membrane lipids [2]. In recent years, the food industry has increasingly marketed foods containing sprouted seeds or flours made from them; once used only in bread, they are now found in tortillas, cereals, cookies, crackers, muffins, snacks, bars, granola, side dishes, and salads [13].

Germination of a seed starts with the uptake of water and cell matrices becoming fully hydrated. After the activation of the hydrolytic enzymes, major mobilization of reserve material occurs and the freed sugars, amino acids, and lipids then provide fuel for the developing root and shoot in the germ [5]. The consumption of the reserve components by the developing embryo during sprouting leads to respiratory losses and thus to a decrease in seed dry matter, which is in accordance with our observations. 

The results of TPC and antioxidant activity assays were all expressed as µmol Trolox equivalent (TE)/g in dry weight (DW) of sample to enable direct comparison of the results for FC, DPPH, and ABTS assays. According to our results, the TPC contents of *Triticum* seeds have been clearly underestimated in the literature without including the bound phenolics. The types of extraction and solvent solution, the number of extraction steps adopted for the extraction of phenolic compounds, and the cleanup methods are all important factors in their recovery. Furthermore, the increase in matrix viscosity promoted by the alkali addition necessary for alkaline hydrolysis of bound phenolics may seriously hinder their extraction [2]. Direct comparison of our results with the literature is therefore difficult, since the absolute value is also affected by genetic and environmental factors. However, our findings are in good agreement with previous work [14], in which extractable phenolics for three different varieties of wheat seed increased by 616.1%, 580.0%, and 442.7% after 4 days of germination. In the cited study, bound phenolics occupied approx. 75% of the total phenolic contents in the raw seeds. Although the content of bound phenolics also increased significantly during the germination process, the proportion of free phenolics to TPC increased steadily in all wheat cultivars, eventually reaching approx. 50%. According to the authors, those results showed that the phenolic composition and distribution were greatly changed by the germination process. Similar findings were observed for spelt, but it is worth mentioning that biosynthesis of phenolics during germination can be additionally stimulated by abiotic stress (salt and sugar osmotic stress, temperature, mechanical damage, drought), as determined in our previous publication [6]. Extractable phenolics are usually synthesized in the intracellular endoplasmic reticulum and stored in vacuoles, while insoluble bound phenolics are formed by the transportation of soluble phenolics to the cell wall and conjugated with cell wall macromolecules through ester and glycosidic bonds, thus contributing to cell wall formation. According to the literature [15], three events occur in relation to the metabolism of phenolic compounds during seed germination. First, the synthesis of natural phenolic compounds starts from glucose or aromatic amino acids that can occur during seed germination. With the oxidative pentose phosphate, glycolytic, and shikimate pathways, aromatic amino acids such as phenylalanine can be produced and transformed into phenolic acids in the cytosol. Phenolic acids then convert into flavonoids, stilbene, and coumarin in the endoplasmic reticulum. These phenolic compounds can be further polymerized or bonded with macromolecular nutrients, such as polysaccharides, proteins, and lipids, thereafter being stored in cell walls or vacuoles. Second, macromolecular nutrients are decomposed by enzymes, which results in the release of phenolics from their bound form. Third, phenolics are consumed to scavenge free radicals or work as intermediates of signal compounds. According to the above, we expected a gradual decrease in bound phenolics (not confirmed in our practical work) and subsequent increase in extractable phenolics in seed without germ (confirmed in our study). In the corresponding germ, we expected to determine only a small increase in extractable phenolics (lower increment despite considerable synthesis of new molecules due to their transformation into the bound form and their activity against oxidative stress) and gradual enlargement of the bound fraction (due to the incorporation of simple phenolics in the cell wall building blocks), but not all our results confirmed our predictions. Moreover, the seed species is an important factor, determining complex biochemical reactions that occur during the germination procedure. Surprisingly, in a study performed by Benincasa et al. [16], bigger differences were observed between two spelt cultivars (cvs. Pietro and Giuseppe) than between spelt (cv. Pietro) and soft wheat (cv. Orso) in terms of total phenolics.

The antioxidant activity of phenolics is often quantified in vitro using high purity compounds, but it has no absolute value. When analyzing their antioxidant contribution in real systems, values will vary due to the presence of other compounds, resulting in significantly lower or higher values. Macromolecules present in the food matrix (carbohydrates, lipids, and proteins) can interact with phenolics through hydrogen bonds and electrostatic and van der Waals interactions, which reduces both their antioxidant activity and their bioaccessibility and bioavailability [17]. However, two analytical methods were adopted to estimate the antioxidant capacity of our extracts. The DPPH analysis indicated a similar overall trend as that observed for the ABTS assay, with the exception of extractable antioxidants obtained from seed: in general, the bound antioxidants were predominant in the seed, while their concentration in the germ was more comparable to that of extractable antioxidants. In addition, a longer germination time did not result in increased antioxidant content, regardless of its form or location. On the other hand, the reactivity of samples against ABTS^•+^ radicals was determined at a higher level than against DPPH^•^ radicals. These results are consistent with another comparative study performed on primitive and modern wheat species [18], in which differences in scavenging capacities between DPPH and ABTS assays were between 6- and 10-fold. Different results on the same samples, analyzed using different assays, can be explained by the composition and concentration of phenolics, which differ between different parts of the sprouted seed and between *Triticum* species (as presented on Figure 3 and Figure 4), as well as in their reactivity toward different radicals. Whereas the reaction between DPPH^•^ radicals and antioxidants is achieved through a hydrogen atom and electron transfer mechanisms, resulting in a non-radical DPPH-H formation, the mechanism of action of the ABTS assay is a mixed hydrogen atom and single electron transfer involving a reduction of generated ABTS^•+^ radicals by the antioxidants present [17]. Moreover, the highest reactivity towards DPPH^•^ radicals was reported for compounds with more than one hydroxyl group (e.g., caffeic acid), followed by monophenols with methoxy (e.g., sinapic, ferulic) or alkyl (e.g., butylated hydroxytoluene) substituents. Compared to DPPH^•^ radicals, ABTS^•+^ reacts much better with monophenols (e.g., ferulic acid, *p*-coumaric acid). Due to the high prevalence of ferulic and *p*-coumaric acid in our extracts, ABTS^•+^ scavenging activity was expected to be higher than that of DPPH^•^. However, not only can the phenolic composition and amount have a significant influence on the obtained results, but also the reaction conditions (e.g., reaction mechanism, time, medium) and data interpretation [19]. Phenolic acids are the most important and largest group of antioxidants in terms of occurrence in cereal seeds. One of the most abundant phenolic compounds in wheat seeds is ferulic acid, which accounts for 90 percent of the total polyphenolics [2]. Over 97% of the ferulic acid in 11 different wheat varieties was found to be bound, and concentrated in the aleurone, pericarp, and embryo cell walls [20]. Our results indicated that germination significantly improves the content of extractable and bound phenolic acids in germinated seed and germ compared to the non-germinated seeds. The germination time and the type of seed (wheat or spelt) did not show any trend in terms of the content of individual extractable and bound phenolic acids. Germ contained a significantly higher amount of monitored phenolic acids than the corresponding germinated seed. Studies [6,21] have usually reported the content of phenolic acid in whole germinated seeds (seed + germ), while our study showed for the first time how phenolic acids are distributed between the germ and the rest of the germinated seed.

### 3.2. Bread Enriched with Germinated Flour

According to previous studies, germinated flour can be used in bread-making recipes to improve the quality of bread from the nutritional and technological points of view [22]. Our results suggest that, in order to obtain bread samples of good quality, the recommended levels of germinated spelt or wheat flour addition in the bread recipe should be up to 5%. A 15% germinated spelt flour incorporation in bread enhanced the extractable TPC and antioxidant activities the most, while it negatively influenced the sensory properties of the bread. A doubled amount of added flour did not further improve the TPC, DPPH, or ABTS values of the prepared breads, in part because food processing can alter the properties of antioxidants through exposure to oxygen, light, temperature, and variations in pH, which ultimately alters the molecular structure of phenols [17]. Bread with 5% germinated spelt flour improved the extractable *p*-coumaric, *trans*-ferulic, and *cis*-ferulic acids the most, while bread with 30% germinated spelt or wheat flour most improved the bound *p*-coumaric, *trans*-ferulic, and *cis*-ferulic acids. Although breads with a higher addition of germinated flour generally had higher TPCs and better antioxidant activity, they were sensorily unacceptable and could not therefore be launched on the market. On the other hand, breads with the addition of 5% germinated spelt or wheat flour showed improved specific volume and, in terms of appearance, aroma, and color, were preferred to other breads. This result might be because, with germinated flour, proteolytic and amylolitic enzymes and soluble compounds are also present in the dough formulation, affecting the technological function of gluten and wheat starch in the bread system [23]. To launch a product on the market, therefore, only breads with 5% germinated spelt or wheat flours were appropriate. In particular, the addition of 30% germinated flour showed a detrimental effect on bread quality (reduction in specific volume, increase in browning, increase in crumb stickiness, and a bitter aftertaste). On the other hand, bread enriched with a higher amount of germinated flour is a rich source of phytochemicals, which can have a positive effect on human health [24]. It is therefore worth considering the inclusion of germinated seeds in bakery products in which gluten and starch do not play such a key role as in bread.

An increase in enzyme activity during germination (e.g., amylase) positively affected the volume of the breads if the amount of added germinated flour did not exceed 5%. Dough development increased, as well as the leavening rate, probably due to the higher presence of simple sugars (i.e., maltose, sucrose, and D-glucose) in sprouted spelt seeds [25], which are useable by yeasts for CO_2_ production. Various studies have correlated this result with a higher α-amylase activity in germinated flour, causing a decrease in the viscosity of the dough, allowing greater gas cell expansion. A huge decrease in specific volume of the bread occurred when 30% germinated flour was added, because the activity of proteases is limited during the germination period, possibly due to the high activity of amylase, which could affect the gluten network [26]. 

Color changes may be attributed to the chemical composition of the germinated flour, the effects of enzymes on germinated flour pigments during fermentation treatment, and a Maillard reaction during the hydrothermal process. The color of the bread crust decreased in luminosity with an increased addition of germinated spelt or wheat flour. The increased addition also resulted in bread samples with a darker crust and a reddish tint. The color change was probably due to the increase in amylase content, which hydrolyses starch and thus increases reducing sugars [27]. Reducing sugars are one of the most important compounds inducing a Maillard reaction, in addition to amino compounds. Compared to the breads without added germinated flour, it is obvious that sugars significantly stimulated the browning reaction. During bread production, a series of complex chemical reactions take place (enzymatic production of sugars, starch gelatinization, caramelization, protein denaturation and coagulation, inactivation of enzymes, Maillard reactions), which play a crucial role in determining the quality characteristics of bread (sensory properties, nutritional value, antioxidant activity). Enzymatic and fermentation reactions affect the flavor of the bread crumb, while heat reactions mainly influence the flavor of the bread crust [28]. 

According to one study [29], the addition of germinated pea flour decreased the loaf volume of bread samples. However, in another study [30], it was shown that the addition of 10% germinated chickpea flour improved the characteristics, while the addition of 20% germinated chickpea flour decreased the bread volume. In addition, the color of the bread samples was not affected by a 10% addition but was significantly affected by a 20% addition. In general, studies have shown a negative influence of a high addition (higher than 10%) on the quality of bread [31], while an addition of up to 3% germinated soy flour has a positive effect. The positive effect was due to enzymes from the germinated flour (lipoxygenase, amylase, lipase, α-galactosidase).

## 4. Materials and Methods

### 4.1. Materials

Wheat (*Triticum* aestivum cv. Chimaboo) and spelt (*Triticum* spelta cv. Ostro) seeds were provided by the local milling industry. Seeds were harvested in Slovenia and stored at 1 °C under darkness until used for analyses. Refined wheat flour (type 500) was obtained from a local store. Folin–Ciocalteu reagent, methanol (99.9%), and sodium dihydrogen phosphate dihydrate were obtained from Merck (Darmstadt, Germany). ABTS reagent, DPPH reagent, hydrochloric acid, manganese (VI) oxide, sodium carbonate, and Trolox were purchased from Sigma-Aldrich (Steinheim, Germany). Sodium hydroxide was purchased from Honeywell Fluka (Seelze, Germany). All of the reagents were of analytical quality. Ultrapure water (MiliQ, Millipore, Burlington, MA, USA) was used for the preparation of solutions.

### 4.2. Germination Performance

The seed (wheat, spelt) samples were carefully cleaned and freed from foreign materials. After a 2 h steeping process at 20 °C (20 g seed, 40 mL of water), the seeds were placed in hot water (47 °C) for 5 min to reduce bacterial and fungal growth. Drained seeds were then placed on moist filter paper in uncovered glass petri dishes. The total amount of water used was 10 mL. Spelt and wheat seeds were allowed to germinate in a germination chamber at 20 °C and atmospheric relative humidity of 98% under darkness. Samples were sprinkled with water (2 mL twice daily) to ensure sufficient water content for synchronized germination. Samples were taken after 48 h and 96 h of biological activation. Untreated (non-germinated) seeds were used as control. Germs and roots (hereinafter referred to as germ) were withdrawn from the remaining germinated seeds (hereinafter referred to as germinated seed). Samples were frozen in liquid nitrogen, freeze dried, and ground to a fine powder using a porcelain mortar for germ or a seed grinder (MKM6000, Bosch^®^, Gerlingen, Germany) for germinated seed. Samples were prepared for the extraction procedure described below. In parallel, an aliquot of milled germs and milled seeds was used for water content determination (by oven drying at 105 °C for 5 h).

### 4.3. Spelt and Wheat Germinated Seeds Flour Preparation

The flour of germinated spelt and wheat seeds was prepared as follows: after 72 h of germination process (described above), seeds were subjected to drying for 12 h at 60 °C, with the aim of reducing their water content to the comparable amount of water that is characteristic of wheat flour used. Dry seeds were ground using a laboratory seed mill (IKA^®^ A11 basic, Staufen, Germany).

### 4.4. Bread Making

In addition to 1000 g of wheat flour, the basic recipe for control bread consisted of 700 mL of water, 7.5 g of instant bakery yeast, 19 g of salt, and 25 mL of sunflower oil. The ingredients were mixed in spiral mixer for 3 min at a lower speed, then for a further 12 min at a higher speed. The dough was allowed to rest for 30 min, after which the dough was divided and molded into round pieces, before it was transferred to a proofing chamber with a temperature of 30 °C and a relative humidity of 85%, for 45 min. After rising, the models with dough were moved to a preheated oven. Baking started with a dosage of 0.5 L of 180 °C hot steam. For the first 5 min the temperature was 230 °C. The bread was baked at 190 °C for a further 20 min and at 200 °C for the last 4 min. The bread was cooled within 2.5 h at room temperature. The recipe for the two control breads (W0, S0) was the same, with only the baking time differing. The control breads were baked at the same time as the corresponding breads enriched with germinated flour. Depending on the type of bread to be made, 5%, 15%, or 30% of commercial white flour was replaced with a relevant proportion of germinated seed flour from wheat (W5, W15, W30) or spelt (S5, S15, S30). All the other ingredients in the recipe and bread-making protocol were the same as for the control. After cooling, some technological and sensory quality parameters of the pan bread were evaluated, while for the chemical analysis, the bread samples were first ground and lyophilized (−50 °C, 30 mTorr) to a moisture level <5%.

### 4.5. Preparation of Crude Extracts of Extractable and Bound Phenolics 

A ground sample (1 g of seed with germ removed/0.2 g of germ/1 g of homogenized and lyophilized bread) was extracted with 9 mL of methanol on a Vibromix shaker (Tehtnica Železniki, Benedikt, Slovenia) at 200 rpm for 2 h. The supernatant and the sediment were separated after centrifugation (10 min, 8000 rpm) and the supernatant was immediately filtered through a 0.45 µm filter (Satorious, Göttingen, Germany). The phenolics that partitioned into the methanolic fraction represented crude extractable phenolics. The sediment was redissolved in 20 mL of 2 M NaOH and stirred at room temperature for 4 h; hydrolysis was interrupted by acidification to pH 2 using 6 M HCl. The crude acidified hydrolysates thus obtained represented the fraction of crude insoluble bound phenolics.

### 4.6. Purification of Crude Extracts 

Extraction and purification of the phenolic compounds from crude extracts were carried out according to the following solid phase extraction (SPE) technique procedure. The SPE cartridge (Strata-X RP, St. Charles, IL, USA, 100 mg) was conditioned with 3 mL of methanol and equilibrated with 3 mL of water. Crude methanolic extracts of extractable phenolics were first subjected to drying in a vacuum evaporator until dryness, followed by reconstruction in the same amount of water and filtered (0.45 µm cellulose mixed ester). Meanwhile, crude acidified hydrolysate was applied directly to the SPE cartridge. The cartridges containing 3 mL of sample (crude extractable or bound phenolics) were then washed with 3 mL of water to remove non-phenolic polar constituents and the absorbed compounds were subsequently eluted with 2 mL of methanol (70 vol.%). The purified methanolic extractable and bound fractions thus obtained were used directly for the analysis of total and individual phenolics, as well for evaluation of their antioxidant properties.

### 4.7. Determination of Total Phenolic Content (TPC) 

TPC in the tested samples was evaluated using the Folin–Ciocalteu assay [32] with some modifications. Briefly, 100 µL of appropriately diluted purified extract was supplemented with 1300 µL Milli-Q water, 300 µL freshly prepared diluted Folin–Ciocalteu reagent with water (1:2, *v*/*v*), and 300 µL sodium carbonate solution (20%, *w*/*w*). After 60 min of incubation in the dark at room temperature, the samples were centrifuged and the absorbance was measured in a 1 cm cuvette at 765 nm on a model 8453 Hewlett Packard UV–Visible spectrophotometer (Hewlett Packard, Waldbronn, Germany) at room temperature. The measurements were compared with a standard curve of a Trolox solution (2.0 mM), and TPC was expressed as µmol Trolox equivalents (TE) per g dry weight of the sample (µmol TE/g DW). Milli-Q water was added to the reaction mixture instead of the sample to obtain a blank.

### 4.8. Analysis of the Polyphenol Composition by LC-MS/MS 

Purified methanolic fractions containing phenolics isolated from samples were analyzed using LC-MS/MS to quantify the predominant ferulic and *p*-coumaric acids in the extracts. The LC-MS/MS system consisted of an Agilent Technologies 1100 binary pump (G1312 A) and an autosampler (G1330 B) coupled to a Micromass Quattro Micro mass spectrometer (QqQ) equipped with an electrospray ionizer source (ESI^−^) (Waters, Milford, MA, USA). Reversed-phase HPLC separation was carried out using a Kinetex C18 column (100 × 2.00 mm; 2.6 µm), protected by a Gemini C18 Security Guard cartridge (4.0 × 2.1 mm) (Phenomenex, Torrance, CA, USA). The mass spectrometer was operated in negative ion mode (ESI^−^) with the following parameters: capillary voltage, 3.0 kV; cone voltage, 20 V; extractor, 2 V. The source temperature was 100 °C, the desolvation temperature was 350 °C, the cone gas flow was 30 L/h, and the desolvation gas flow was 350 L/h. The mobile phase components were 0.1% formic acid (A) and acetonitrile (B). The mobile-phase gradient used was: 0–2 min, 10% B; 2 20 min, 10–60% B; 20–21 min, 60–80% B; 21–25 min, 80% B; 25–26 min, 80–10% B; 26–30 min, 10% B. The injection volume was 10 µL and the column temperature was 25 °C. The flow rate of the mobile phase was 0.300 mL/min. The data signals were acquired and processed on a PC using MassLynx software (V4.1 2005; Waters Corporation, Milford, MA, USA). The phenolic acids were identified on the basis of their retention times and MS spectra, and quantified according to their corresponding standards. 

### 4.9. Antioxidant Activity Determined by Using DPPH Assay 

The radical scavenging activity of purified extracts against DPPH^•^ radical was determined in methanol [33]. A 100 µL aliquot of the extract was supplemented with 900 µL methanol and added to a 1000 µL 0.2 mM solution of DPPH^•^. After 60 min of incubation in the dark at room temperature, followed by centrifugation, absorbance was read at 520 nm. A solution of 1 mM Trolox in methanol was used as the standard to establish a standard curve. The absorbance of the DPPH^•^ radical supplemented with methanol without any antioxidants (reference solution) was measured before each test. Milli-Q water was used as a blank for all the readings. DPPH^•^ scavenging activity was expressed as µmol TE/g DW.

### 4.10. Antioxidant Activity Determined by Using the ABTS Assay

The radical scavenging activity of extracts against ABTS^•+^ radical was performed in phosphate buffer solution [34]. The ABTS radical cation (ABTS^•+^) was produced by reacting ABTS stock solution with manganese dioxide as the oxidizing agent. An aliquot of extract (50 µL) was supplemented with 750 µL of water and added to 200 µL phosphate buffer (pH 7.4) and 1000 µL ABTS working solution. The reaction mixture was vortexed. After 60 min of incubation in the dark at room temperature, followed by centrifugation, absorbance was read at 734 nm. A solution of Trolox in methanol (1 mM) was used as the standard to establish a standard curve. The reference solution consisted of ABTS solution and the same amount of water. Water was used as a blank. ABTS^•+^ scavenging activity was expressed as µmol TE/g DW.

### 4.11. Specific Volume 

The specific volume of the bread was determined 3 h after baking by the rapeseed displacement method, AACC method 10-05.01 [35]. The weight of the bread was recorded and the specific volume was calculated as the ratio between the bread volume (mL) and weight (g). 

### 4.12. Color Measurement

The coloration of bread samples was determined by measuring the L* a* b*, where L* represents the luminosity, a* the redness, and b* the yellowness. The analysis was performed using a color-measurement Minolta CR-400 device (Konica Minolta, Kyoto, Japan). The color was measured five times over the entire surface of each loaf of bread (crust)), and the average value was used. The total color change (ΔE) between the control and breads enriched with germinated flour was calculated using the following formula: $\Delta E=\sqrt{{\Delta L}^{2}+{\Delta a}^{2}+{\Delta b}^{2}}$.

### 4.13. Sensory Evaluation

Sensory evaluation was performed by trained panelists 3 h and 24 h after baking. Representative slices (2 cm thick) were numbered and served to each participant to assess the general acceptability of the breads enriched with germinated flour. Acceptability of the bread’s appearance, aroma, taste, texture, color, and odor was evaluated using descriptive sensory analysis. The external appearance of the bread was first evaluated (shape and appearance of the bread; appearance and color of the crust). Then, a slice of bread was cut open and the appearance of the crumb (porosity, color), the elasticity and stickiness of the crumb, the crispness of the crust, the odor, and the taste were evaluated. The individual parameters were described as the intensity of each parameter compared to the control breads “Bread S0” and “Bread W0”: color (lighter/darker), porosity (lower/higher), elasticity (lower/higher), hardness (softer/harder), stickiness (from none to very sticky), aftertaste (from none to strong), bitterness (from none to strong), and overall taste and aroma (less pronounced/stronger).

### 4.14. Statistical Analysis 

The experiment was performed in three replications (each replication in two parallels) and statistical analysis was conducted using SPSS for Windows (Version 21), using one way analysis of variance (ANOVA). The LSD (for germ and corresponding germinated seed) and Duncan post hoc tests (for non-germinated seeds and breads) were used to assess differences between means; differences were considered significant at the *p* < 0.05 level. Multivariate statistical analysis (principal component analysis (PCA)) was performed to interpret the differences in all of the analyzed samples using OriginPro 2015.

## 5. Conclusions

This study demonstrates that, in germinated seed (without germ), the majority of TPC was determined to be bound phenolics, while the extractable form dominated in the germ. Furthermore, germ contained a significantly higher content of monitored phenolic acids than the corresponding germinated seed. These results show that the phenolic composition and distribution were significantly changed by the germination process. Our results also indicate that the germination time and type of seed (wheat or spelt) did not show any trend in terms of the content of individual extractable and bound phenolic acids. Evaluation of bread enriched with germinated flour showed that bread with 5% germinated spelt flour improved extractable *p*-coumaric, *trans*-ferulic, and *cis*-ferulic acids the most, while bread with 30% germinated spelt or wheat flour improved their bound forms the most. The use of germinated spelt or wheat flour as a 5% substitute in the white flour formulation enriched the bread with higher extractable TPC, antioxidant capacity, and individual phenolic acids, and resulted in a product with improved specific volume and preferred appearance, aroma, and color. To launch a product on the market, only breads with 5% germinated spelt or wheat flours were appropriate. The bread-making performance evaluated in terms of biochemical characteristics, specific volume, color, and sensory properties confirms that germinated spelt or wheat flour is a promising and interesting ingredient for the formulation of bakery products, while avoiding the use of enzymatic improvers or malt, with a positive impact on consumer acceptance and facilitating the adoption of a clean label.

## Figures and Tables

**Table 1:** Total phenolic content (TPC) and in vitro antioxidant activities (DPPH^•^ (DPPH) and ABTS^•+^ (ABTS) antioxidant capacity) for the extractable (EXT) and bound (BND) phenolics in the non-germinated and germinated seeds (µmol TE/g DW).

Germination		TPC		DPPH		ABTS	
Time	Seeds	Extractable	Bound	Extractable	Bound	Extractable	Bound
0 h	wheat	1.84 ^a^	31.67 ^b^	0.46 ^a^	3.01 ^b^	1.83 ^a^	29.71 ^b^
	spelt	2.47 ^a^	27.07 ^a^	0.34 ^a^	2.59 ^a^	3.39 ^b^	26.53 ^a^
48 h	wheat	10.42 ^b^	36.01 ^c^	2.15 ^c^	4.05 ^d^	10.19 ^c^	42.64 ^d^
	spelt	10.75 ^b^	30.71 ^b^	1.75 ^b^	3.54 ^c^	10.69 ^c^	36.45 ^c^
96 h	wheat	26.03 ^c^	40.79 ^d^	4.45 ^d^	4.96 ^e^	22.39 ^d^	49.51 ^e^
	spelt	30.64 ^d^	43.93 ^d^	4.87 ^d^	4.83 ^e^	26.91 ^e^	52.72 ^e^

Data are means ± standard deviation, and different letters within the column indicate significant differences (*p* < 0.05; Duncan’s Multiple Range Test).

**Table 2:** Content of three most dominant phenolic acids in wheat and spelt non-germinated seeds and their distribution between seed and germ during germination (µg/g DW).

	*p*-Coumaric Acid		*trans*-Ferulic Acid		*cis*-Ferulic Acid	
Samples	Extractable	Bound	Extractable	Bound	Extractable	Bound
wheat non-germinated	1.43 ± 0.17 ^a^	11.47 ± 0.11 ^A^	11.29 ± 0.56 ^a^	345.67 ± 15.24 ^A^	11.09 ± 0.44 ^a^	155.78 ± 1.97 ^A^
spelt non-germinated	1.37 ± 0.02 ^a^	12.77 ± 0.83 ^B^	10.83 ± 0.15 ^a^	349.60 ± 59.08 ^A^	10.92 ± 0.22 ^a^	122.01 ± 32.31 ^A^
**Seed**						
wheat 48 h	2.15 ± 0.10 ^a^	16.51 ± 1.92 ^A^	16.38 ± 0.84 ^a^	430.75 ± 98.45 ^A^	15.62 ± 0.20 ^a^	136.53 ± 20.36 ^A^
spelt 48 h	2.21 ± 0.07 ^a^	15.93 ± 1.30 ^A^	15.73 ± 0.47 ^a^	387.59 ± 32.43 ^A^	15.29 ± 0.24 ^a^	156.44 ± 36.01 ^AB^
wheat 96 h	2.15 ± 0.13 ^a^	17.23 ± 1.47 ^A^	16.60 ± 0.21 ^a^	469.29 ± 55.95 ^AB^	16.25 ± 0.75 ^b^	155.73 ± 21.38 ^AB^
spelt 96 h	2.18 ± 0.06 ^a^	20.64 ± 1.40 ^B^	16.43 ± 0.36 ^a^	549.74 ± 43.79 ^B^	16.30 ± 0.52 ^b^	186.90 ± 31.50 ^B^
**Germ**						
wheat 48 h	53.24 ± 6.08 ^a^	177.79 ± 14.66 ^A^	391.14 ± 30.25 ^b^	2352.27 ± 171.06 ^A^	392.33 ± 32.60 ^b^	1693.67 ± 47.02 ^B^
spelt 48 h	48.58 ± 3.11 ^a^	187.28 ± 8.96 ^A^	319.57 ± 25.35 ^a^	2342.07 ± 388.21 ^A^	344.36 ± 22.35 ^a^	1317.26 ± 193.42 ^A^
wheat 96 h	54.52 ± 5.52 ^a^	488.45 ± 56.44 ^C^	334.07 ± 47.35 ^ab^	2658.34 ± 231.34 ^A^	307.81 ± 4.94 ^a^	1323.36 ± 167.00 ^A^
spelt 96 h	58.35 ± 9.40 ^a^	384.70 ± 56.79 ^B^	369.40 ± 23.29 ^b^	2624.71 ± 161.35 ^A^	361.43 ± 20.81 ^b^	1829.02 ± 143.22 ^B^

Data are means ± standard deviation. Different letters within the same fraction (extractable or bound) and within the non-germinated seeds/germinated seed/germ indicate significant differences (*p* < 0.05; Duncan’s Multiple Range Test).

**Table 3:** Influence of germinated spelt or wheat flour on phenolic acids (µg/g) profile in enriched and control breads.

	*p*-Coumaric Acid		*trans*-Ferulic Acid		*cis*-Ferulic Acid	
Bread	Extractable	Bound	Extractable	Bound	Extractable	Bound
**S0**	10.48 ± 0.74 ^a^	15.53 ± 1.2 ^a^	0.66 ± 0.18 ^a^	58.17 ± 3.06 ^a^	1.17 ± 0.71 ^a^	59.50 ± 12.63 ^ab^
**S5**	19.89 ± 0.16 ^bc^	20.40 ± 3.95 ^a^	4.83 ± 0.69 ^c^	95.28 ± 10.30 ^a^	3.97 ± 0.78 ^c^	55.69 ± 0.14 ^ab^
**S15**	10.57 ± 0.67 ^a^	53.41 ± 6.08 ^b^	4.05 ± 0.33 ^c^	213.63 ± 42.41 ^b^	2.50 ± 0.64 ^abc^	111.83 ± 12.73 ^c^
**S30**	10.55 ± 0.99 ^a^	79.36 ± 2.56 ^c^	2.98 ± 0.28 ^b^	428.16 ± 16.8 ^c^	3.44 ± 0.68 ^abc^	154.49 ± 1.03 ^d^
**W0**	16.47 ± 0.03 ^b^	15.90 ± 0.03 ^a^	0.65 ± 0.14 ^a^	42.85 ± 8.41 ^a^	1.48 ± 0.82 ^ab^	33.63 ± 7.36 ^a^
**W5**	21.1 ± 3.31 ^bc^	21.70 ± 3.96 ^a^	2.16 ± 0.41 ^b^	65.23 ± 9.41 ^a^	3.72 ± 0.67 ^bc^	87.91 ± 18.91 ^bc^
**W15**	22.00 ± 2.24 ^c^	49.32 ± 1.73 ^b^	3.05 ± 0.43 ^b^	197.88 ± 27.66 ^b^	4.15 ± 0.36 ^c^	183.45 ± 23.57 ^d^
**W30**	17.38 ± 0.63 ^bc^	70.10 ± 12.64 ^c^	2.73 ± 0.21 ^b^	212.98 ± 44.43 ^b^	2.97 ± 0.27 ^abc^	180.84 ± 17.84 ^d^

Data are means ± standard deviation. Different letters within the same fraction (extractable or bound) indicate significant differences (*p* < 0.05; Duncan’s Multiple Range Test). S0: control bread with no added germinated flour; S5: bread with added 5% germinated spelt flour; S15: bread with added 15% germinated spelt flour; S30: bread with added 30% germinated spelt flour; W0: control bread with no added germinated flour; W5: bread with added 5% germinated wheat flour; W15: bread with added 15% germinated wheat flour; W30: bread with added 30% germinated wheat flour.

**Table 4:** Effects of addition of 0%, 5%, 15% and 30% germinated spelt or wheat flour on specific volume and color values of breads.

Bread	Specific Volume (mL/g)	Luminosity (L*)	Browning (100-L*)	Redness (a*)	Yellowness (b*)	Total Color Change (ΔE)
**S0**	2.56 ± 0.07 ^c^	67.67 ± 3.83 ^e^	32.33 ± 3.83 ^b^	9.03 ± 2.24 ^b^	36.71 ± 1.67 ^d^	/
**S5**	2.85 ± 0.10 ^d^	59.72 ± 2.64 ^d^	40.28 ± 2.64 ^c^	11.88 ± 1.47 ^d^	32.78 ± 1.79 ^c^	9.30
**S15**	2.63 ± 0.04 ^c^	56.52 ± 3.22 ^c^	43.48 ± 3.22 ^d^	8.61 ± 0.54 ^b^	26.91 ± 1.50 ^b^	14.85
**S30**	1.75 ± 0.04 ^a^	43.91 ± 1.79 ^a^	56.09 ± 1.79 ^f^	11.41 ± 2.14 ^cd^	21.07 ± 4.36 ^a^	28.54
**W0**	3.10 ± 0.02 ^e^	69.71 ± 1.91 ^f^	30.29 ± 1.91 ^a^	6.64 ± 0.81 ^a^	26.89 ± 1.14 ^b^	/
**W5**	3.83 ± 0.13 ^f^	59.79 ± 3.14 ^d^	40.21 ± 3.14 ^c^	8.41 ± 0.52 ^b^	28.19 ± 1.17 ^b^	10.16
**W15**	3.15 ± 0.07 ^e^	55.64 ± 2.30 ^c^	44.36 ± 2.30 ^d^	8.22 ± 0.69 ^b^	26.33 ± 1.27 ^b^	14.17
**W30**	2.20 ± 0.02 ^b^	46.06 ± 2.69 ^b^	53.94 ± 2.69 ^e^	10.34 ± 1.90 ^c^	23.17 ± 1.63 ^a^	24.20

Data are means ± standard deviation. Different letters within a column indicate significant differences (*p* < 0.05; Duncan’s Multiple Range Test). S0: control bread with no added germinated flour; S5: bread with added 5% germinated spelt flour; S15: bread with added 15% germinated spelt flour; S30: bread with added 30% germinated spelt flour; W0: control bread with no added germinated flour; W5: bread with added 5% germinated wheat flour; W15: bread with added 15% germinated wheat flour; W30: bread with added 30% germinated wheat flour.

## Data Availability

All data are contained in this article.
